# Genotypic Characterization and Biofilm Production of Group B Streptococcus Strains Isolated from Bone and Joint Infections

**DOI:** 10.1128/spectrum.02329-21

**Published:** 2022-03-31

**Authors:** Marion Lacasse, Anne-Sophie Valentin, Stéphane Corvec, Pascale Bémer, Anne Jolivet-Gougeon, Chloé Plouzeau, Didier Tandé, Laurent Mereghetti, Louis Bernard, Marie-Frédérique Lartigue

**Affiliations:** a Université de Tours, INRAE, ISP, Tours, France; b Centre Hospitalier Universitaire de Tours, Service de Bactériologie, Virologie et Hygiène Hospitalière, Tours, France; c University Hospital Center of Nantes, Bacteriology Department, Nantes University, Nantes, France; d University of Rennes, INSERM, University Hospital of Rennes, NUMECAN Institute (Nutrition Metabolisms and Cancer), Rennes, France; e Bacteriology-Hospital Hygiene Department, University Hospital of Poitiers, Poitiers University, Poitiers, France; f Bacteriology-Hospital Hygiene Department, University Hospital of Brest, Brest University, Brest, France; g Centre Hospitalier Universitaire de Tours, Service de Maladies infectieuses, Tours, France; Ohio State University

**Keywords:** biofilm, group B *Streptococcus*, bone and joint infections, MLST, adhesins, pili

## Abstract

Bone and joint infections (BJI) represent the second cause of invasive Group B Streptococcus (GBS) infections. Biofilm formation plays a major role in BJI. This study’s aim was to analyze the genetic features and biofilm production of GBS strains. In six French laboratories, 77 GBS strains isolated from BJI and 57 strains from vaginal human colonization (Hcol) were characterized and compared by Multi-Locus Sequence Typing (MLST). PCR was used to search for the adhesins (*bsaB*, *lmb*, *scpB*, *fbsA*, *fbsB*, *hvgA*, *bibA*, *bca*, *srr-1,* and *srr-2*) and Pilus Islands (PI) related genes (PI-1, PI-2a, PI-2b). Biofilm production was studied by crystal violet assay. Strains were categorized into three groups, based on Specific Biofilm Formation (SBF) values defined as: weak, moderate, or strong producers. Molecular study revealed three major clonal complexes (CC) in BJI strains: CC1 (42%), CC23 (22%) and CC10 (14%). Several associations between CC and adhesin/pili were identified: CC1 with *srr2,* PI-1 + 2a; CC10 with *srr-1, bca,* PI-1 + 2a; CC17 with *fbsB*, *hvgA, srr-2,* PI-1+PI-2b; CC19 with *bibA*, *srr-1*, PI-1 + 2a; CC23 with *fbsB*, *bibA*, *srr-1*, PI-2a. The biofilm production was significantly different according to CC, adhesins and pili gene detection. CC10, CC23 and strains harboring *fbsB* produce more biofilm than CC1, PI-1 + 2a (independently). Finally, SBF values were significantly stronger for Hcol strains rather than for BJI strains (76% *versus* 40%). This study revealed that Hcol strains appeared to produce stronger biofilm than BJI strains, though they belonged to similar CCs and had the same adhesin and pili content.

**IMPORTANCE** Bone and joint infections (BJI) are pathologies that can be life-threatening and result in compromised functional prognosis for patients. Relapses are common and often related to biofilm formation. Group B streptococci (GBS) BJI increased since the last decade. However, few data are available on this subject in the literature. Our study aims to highlight genotype and biofilm production of GBS isolates from BJI. Seventy-seven GBS strains isolated from BJI and 57 from asymptomatic human vaginal colonization were characterized by multilocus sequence typing (MLST), adhesins content, nature of the pili and the ability to form biofilm. Our results revealed that vaginal human colonization strains produced stronger biofilm than BJI strains, despite belonging to the same phylogenetic lineage and having the same adhesin and pili content.

## INTRODUCTION

The rate of group B Streptococcus bone and joint infections (BJI) has increased substantially in the past decade (1). Streptococcal prosthetic-joint infections (PJI) can be life-threatening and functionally challenging for patients, with a high risk of treatment failure and long-term morbidity (2–4). This risk has been associated with frequent progression to chronicity or recurrence (4, 5). The pathophysiology of BJI is different according to the presence or absence of orthopedic device (5). In the case of the device is present in the joint, the chronicity is facilitated by bacterial adherence and biofilm formation (6, 7). The most common bacterium involved in BJI remains Staphylococcus. *Streptococci* matter for only 17% of BJI and Streptococcus agalactiae (named also Group B Streptococcus or GBS) represents 4% of them (8). Few data in the literature concern GBS BJI (1, 8). GBS is a commensal bacteria of the genital and gastrointestinal tract found in 15–32% of healthy adults (9). Multi-Locus Sequence Typing (MLST) allows the classification of the majority of GBS strains associated with human infections into five major clonal complexes (CCs) (9). CC17 and CC19 GBS strains have a major role in invasive neonatal diseases (10). In contrast, CC1, CC10 and CC23 strains cause invasive diseases for nonpregnant adults (11). GBS presents virulence factors leading to either colonization and/or infection. Among them, there are surface-associated adhesins involved in a fundamental step of the pathogenicity of BJI. These include fibrinogen-binding proteins (FbsA, FbsB, Srr1, and Srr2) (12, 13), the hypervirulent GBS adhesin (HvgA) (14), laminin-binding protein (Lmb) (12), C5a peptidase (ScpB) (12), bacterial surface adhesin of GBS (BsaB) (15), alpha C protein (encoded by *bca* gene) (16), and the immunogenic bacterial adhesin (BibA) (17). Other biofilm factors have been reported, such as pili protruding on the bacterial surface and facilitating host-cell attachment. The pili are the result of three pilin proteins assembly: the pilus shaft backbone protein (PilB), the pilus tip (PilA, the pilus-associated adhesin), and the pilus base (PilC). Pilus Islands (PI)-1, PI2a and PI2b are variants of pili. PIs are found on two genomic islands. All GBS have one or a combination of variants of these PIs, with PI-2a and PI-2b being mutually exclusive. Strains with only PI-2a pili form significantly more biofilm than strains with PI-1 and PI-2b pili (18, 19).

The role of biofilm in GBS BJI is unknown, the main objective of the study is to determine particular genotypes of GBS strains involved in BJI.

## RESULTS

### Clinical features of bone and joint infections by GBS.

From January 2010 to December 2016, 77 cases of GBS BJIs were identified. Fifty-four % of patients were male, and the median age was 70 years. Sixty-three patients (82%) had at least one comorbidity. Most frequently reported comorbidities were arterial hypertension (46%), overweight (37%), diabetes mellitus (26%), and heart failure (25%). Sixteen % of patients reported a history of BJI. The infection occurred on an orthopedic device in 66% of patients, mainly on hip (47%) and knee (40%) (Table 1).

**TABLE 1:** Clinical characteristics of 77 patients with GBS bone and joint infections^*a*^

Clinical characteristics	No. of patients (%)
Gender, male	41/77 (54)
Age, yrs	70 (20-95)
Comorbidities	
No comorbidity One comorbidity Two comorbidities > Two comorbidities Hypertension Overweight (Body Mass Index > 30) Diabetes mellitus Heart failure Smoker Vascular insufficiency History of GBS BJI Cancer/Hemopathy Pulmonary disease Systemic or inflammatory disease Stroke/dementia Liver failure Kidney failure	14/77 (18) 26/77 (34) 21/77 (27) 16/77 (21) 35/76 (46) 21/57 (37) 20/77 (26) 19/76 (25) 12/71 (17) 12/75 (16) 12/76 (16) 9/77 (12) 8/77 (10) 8/77 (10) 6/77 (8) 5/75 (7) 4/77 (5)
Immunosuppressive therapy	3/75 (4)
Anti-inflammatory drugs	3/74 (4)
Localization of infection total and in orthopedic devices	
Hip/material Knee/material Foot/material	29/77 (38)/22/47 (47) 24/77 (31)/19/47 (40) 6/77 (8)/1/47 (2)
Spine/material Leg/material	4/77 (5)/1/47 (2) 4/77 (5)/2/47 (4)
Wrist/material	2/77 (3)/1/47 (2)
Ankle/material Pelvis Hand	2/77 (3)/1/47 (2) 1/77 (1) 1/77 (1)
Shoulder	1/77 (1)
Unknown	3/77 (4)
Delayed of infection (joint age)	
Early < 1 mo Delayed between 1 and 12 mo	7/50 (14) 10/50 (20)
Late > 12 mo	33/50 (66)
	
Clinical signs	
CRP > 10 mg/mL Pain Functional impotence Fever	59/60 (100) 57/67 (85) 49/63 (78) 45/64 (70)
Erythema Presence of purulence	40/67 (59) 37/65 (57)
Dirty scar	17/54 (31)
Bacteremia or endocarditis	9/71 (13)

a Results are expressed as median (minimum; maximum) or n (%).

In most of the cases, the infection occurred at least 1 year postoperatively (66%). Main clinical reported signs were fever, functional impotence, pain, and elevated serum C reactive protein (CRP) (71, 79, 86 and 100%, respectively) (Table 1).

Ten % of patients with an orthopedic device experienced a treatment failure.

### Genotypic characterization of BJI and Hcol strains.

Seventy-seven strains have been isolated from joint fluid or deep surgical site sampling and 57 from the vaginas of asymptomatic women at prenatal screening (week 35–37). Using the MLST, 36 different STs were identified. Most frequent CCs in BJI were CC1 (42%), CC23 (22%) and CC10 (14%). No significant difference in CCs distribution was observed between BJI and Hcol strains (Table 2).

**TABLE 2:** Distribution of adhesins and pili between BJI and Hcol strains and between the different CCs

	All CCs (%)			CC1 (%)			CC10 (%)			CC17 (%)			CC19 (%)			CC23 (%)			Other CCs (%)^*b*^		
Genes	BJI *n* = 77	Hcol *n* = 57	Total *n* = 134	BJI *n* = 32	Hcol *n* = 17	Total *n* = 49	BJI *n* = 11	Hcol *n* = 13	Total *n* = 24	BJI *n* = 6	Hcol *n* = 6	Total *n* = 12	BJI *n* = 8	Hcol *n* = 5	Total *n* = 13	BJI *n* = 17	Hcol *n* = 11	Total *n* = 28	BJI *n* = 3	Hcol *n* = 5	Total *n* = 8
*fbsB*	25 (32)	17 (30)	42 (31)	2 (6)	1 (6)	3 (6)	0	0	0	6 (100)	6 (100)	12 (100)^*a*^	0	0	0	17 (100)	10 (91)	27 (96)^*a*^	0	0	0
*hvgA*	6 (8)	6 (11)	12 (9)	0	0	0	0	0	0	6 (100)	6 (100)	12 (100)^*a*^	0	0	0	0	0	0	0	0	0
*bibA*	27 (35)	18 (32)	45 (34)	2 (6)	1 (6)	3 (6)	0	2 (15)	2 (8)	0	0	0	8 (100)	5 (100)	13 (100)^*a*^	17 (100)	10 (91)	27 (96)^*a*^	0	0	0
*bca*	16 (21)	17 (30)	33 (25)	3 (9)	1 (6)	4 (8)	10 (91)	13 (100)	23 (96)^*a*^	0	0	0	1 (13)	0	1 (8)	0	1 (9)	1 (4)	2 (67)	2 (40)	4 (50)
*srr−1*	60 (78)	51 (89)	111 (83)	24 (75)	17 (100)	41 (84)	10 (91)	13 (100)	23 (96)^*a*^	0	0	0	7 (88)	5 (100)	12 (92)	16 (94)	11 (100)	27 (96)^*a*^	3 (100)	5 (100)	8 (100)
*srr−2*	6 (8)	6 (8)	12 (9)	0	0	0	0	0	0	6 (100)	6 (100)	12 (100)^*a*^	0	0	0	0	0	0	0	0	0
*PI−1 + 2a*	51 (66)	36 (63)	87 (65)	31 (97)	14 (82)	45 (92)^*a*^	11 (100)	13 (100)	24 (100)^*a*^	0	0	0	8 (100)	5 (100)	13 (100)^*a*^	1 (6)	3 (27)	4 (14)	0	1 (20)	1 (13)
*PI−2a*	18 (23)	14 (25)	32 (24)	0	3 (18)	3 (6)	0	0	0	0	0	0	0	0	0	16 (94)	8 (73)	24 (86)^*a*^	2 (67)	3 (60)	5 (63)
*PI−1 + 2b*	8 (10)	7 (12)	15 (11)	1 (3)	0	1 (2)	0	0	0	6 (100)	6 (100)	12 (100)^*a*^	0	0	0	0	0	0	1 (33)	1 (20)	2 (25)

a *P* < 0.05. Gray shading is the total number of strains.

b Other CCs are CC4 (*n* = 1), CC7 (*n* = 3), CC22 (*n* = 1), CC130 (*n* = 1), and CC388 (*n* = 2).

The genes encoding 10 adhesins and three pili islands genes (PI-1, PI-2a and PI-2b) have been searched by PCR (Table 2). Genes encoding BsaB, Lmb, ScpB and FbsA proteins were detected in each strain regardless of their origin. The *srr-1* gene was amplified in 82%, the *bib*A gene in 34%, the *fbsB* gene in 31% and the *bca* gene in 25% of the 134 strains (Table 2). Only 9% of the 134 strains possessed the *hvgA* and *srr-*2 genes. The comparison of adhesins distribution between BJI and Hcol strains within the same CC showed no significant difference, whereas the comparison of adhesins/pili distribution between CCs (regardless of strain origin) showed significant associations (Table 2). Indeed, the *bca* gene was detected in 96% of CC10 strains whereas only 9% (10/110 strains) of all other CCs harbored this adhesin (*P* < 0.001). In the same way, the *srr-2* and *hvgA* genes were only found in CC17 strains (*P* < 0.001). Conversely, none of CC17 strains possessed the *srr-1* gene (*P* < 10^−13^); 196% of the strains belonging to CC17 and CC23, respectively, possessed the *fbsB* gene (*P* < 0.001). The *bibA* gene was detected in each CC19 strain and in 96% of CC23 strains (*P* < 0.001).

The nature of the pili was studied in 134 strains (Table 2). At least, one genomic island of pili was amplified in each strain. PI-1 was always associated with another genomic island. No significant difference was found in the pili distribution between Hcol and BJI strains. The most frequent pili combination was PI-1 associated with PI-2a found in 65% of 134 strains. PI-2a alone was found in 24% of 134 strains, mostly CC23 strains (86%) (*P* < 0.001). The combination PI-1 and PI-2b was found in only 11% of the strains, mainly CC17 strains (100%) (*P* < 0.001). An association between pili and CC was found (Table 2). The association of PI-1 and PI-2a was significantly associated with the CC1 (92%), CC10 (100%) and CC19 (100%) (*P* < 0.05).

### Origin and genotype of strains are predictive of biofilm phenotypes.

Biofilm production of 134 strains was studied by evaluating the proportions of bacteria sticking to the polystyrene plate and was classified into three categories according to the SBF: weak (SBF < 1.5), moderate (SBF between 1.5 and 3) or strong (SBF> 3) producers.

Biofilm production varied significantly according to the origin of the strains. Seventy-five % (43/57) of Hcol strains were moderate or strong biofilm producers whereas 60% (46/77) of BJIs strains were not (*P* < 0.001). A difference in biofilm production was also observed according to the CC of the strains (*P* < 0.0001); 55 % of CC1 strains are classified in weak biofilm producers. They represent 45% of all strains with SBF < 1.5 (*P* < 0.0001). Conversely, an SBF between 1.5 and 3 was obtained in 31% of the CC23 strains and 46% of the CC10 strains were strong producers. Seventy-eight and 79% of CC23 and CC10 strains, respectively, were moderate or strong biofilm producers (*P* < 0.01) (Table 3). Finally, biofilm production did not vary significantly among the strains exhibiting different islands of pili whereas it varied significantly according to the FbsB and BibA adhesins. Thirty-nine and 41% of strains that possessed *fbsB* and *bibA* gene, respectively, had an SBF >1.5 (*P* < 0.05).

**TABLE 3:** Biofilm production of strains according to their CCs, pili, and adhesins

CCs/pili/adhesins	SBF < 1.5 weak biofilm producers (%)			1.5 < sBF < 3 moderate biofilm producers (%)			SBF > 3 strong biofilm producers (%)		
	BJI *n* = 46	Hcol *n* = 14	Total *n* = 60	BJI *n* = 16	Hcol *n* = 26	Total *n* = 42	BJI *n* = 15	Hcol *n* = 17	Total *n* = 32
CC1, *n* = 49	24 (52)	9 (64)	33 (55)^*a*^	6 (38	6 (23)	12 (28)	2 (13)	2 (12)	4 (13)
CC10, *n* = 24	4 (9)	1 (7)	5 (8)	4 (25)	9 (35)	13 (31)^*a*^	3 (20)	3 (18)	6 (19)
CC17, *n* = 12	6 (13)	0	6 (10)	0	3 (12)	3 (7)	0	3 (18)	3 (9)
CC19, *n* = 13	6 (13)	2 (14)	8 (13)	2 (12)	2 (8)	4 (10)	0	1 (6)	1 (3)
CC23, *n* = 28	6 (13)	0	6 (10)	2 (12)	5 (19)	7 (17)	9 (60)	6 (36)	15 (46)^*a*^
Other CCs^*b*^, *n* = 8	0	2 (14)	2 (3)	2 (12)	1 (4)	3 (7)	1 (7)	2 (12)	3 (9)
PI-1 + PI−2a, *n* = 87	34 (74)	9 (64)	43 (72)	13 (81)	20 (56)	33 (79)^*a*^	4 (27)	7 (41)	11 (34)
PI−2a, *n* = 32	6 (13)	4 (29)	10 (17)	2 (12)	3 (12)	5 (12)	10 (67)	7 (41)	17 (53)^*a*^
PI−1 + PI−2b, *n* = 15	6 (13)	1 (7)	7 (12)	1 (6)	3 (12)	4 (10)	1 (7)	3 (18)	4 (13)
*fbsB*, *n* = 42	12 (26)	1 (7)	13 (22)	3 (19)	8 (31)	11 (26)	10 (67)	8 (47)	18 (56)^*a*^
*hvgA*, *n* = 12	6 (13)	0	6 (10)	0	3 (12)	3 (7)	0	3 (18)	3 (9)
*bibA*, *n* = 45	12 (26)	3 (21)	15 (25)	6 (38)	8 (31)	14 (33)	9 (60)	7 (41)	16 (50)^*a*^
*bca*, *n* = 33	6 (13)	4 (28)	10 (17)	7 (44)	9 (35)	16 (38)	3 (20)	4 (23)	7 (22)
*srr−1*, *n* = 111	35 (76)	14 (100)	49 (82)	14 (88)	23 (88)	37 (88)	11 (73)	14 (82)	25 (78)
*srr−2*, *n* = 12	6 (13)	0	6 (10)	0	3 (12)	3 (7)	0	3 (18)	3 (9)

a *P* < 0.05. Gray shading is the total number of strain.

b Other CCs are CC4 (*n* = 1), CC7 (*n* = 3), CC22 (*n* = 1), CC130 (*n* = 1), and CC388 (*n* = 2).

## DISCUSSION

This work constitutes the first multicenter study describing the clinical and microbiological features of 77 cases of GBS BJIs compared to 57 Hcol strains. Results suggest that particular genotypes of GBS strains promote the biofilm formation ability. Thus, GBS Hcol strains seem to produce more biofilm than GBS BJI strains.

In this study, GBS isolates that are responsible of BJIs belong to three predominant CCs, accounting for 68% of all isolates, namely, CC1, CC10, and CC23. Therefore, unlike the association described between neonatal infections and the hypervirulent clone ST-17, BJIs are not associated with a particular and emerging GBS clone (10).

With the method used and the conditions developed, the results show that only strains belonging to CC10 and CC23 were strong biofilm producers, and strains belonging to CC1 were not. Conversely, Maeda et al. (20) did not observe differences in the distribution of main CCs (CC1, CC10, CC19, and CC23) between the producer and nonproducer populations, but the number of strains in their study was smaller than in ours.

CC23 strains contained the *bibA*, *fbsB* and variant genes of pili PI-2a, and CC10 strains contained *bca*, *srr-1* and genes of pili PI-1 + 2a. These results are in accordance with those of the literature (19, 21–23).

There was no significant difference between Hcol and BJI GBS strains in their distribution within CC or also in their gene content encoding both adhesins and pili genomic island. However, although they belong to similar CCs and they have the same adhesin and pili content, GBS Hcol strains appear to produce more biofilm than GBS BJI strains. Thus, other factors such as bacteriophages could be involved in biofilm formation. Indeed, the ROSA type prophage in S. aureus is associated with the high biofilm formation and colonization. It influences the bacteria’s virulence profile. Moreover, in BJI, ROSA-like abolishes the S. aureus ability to replicate intracellularly and improve survival in infected osteoblasts (24).

While biofilm is a complicating and relapsing factor in BJI especially in S. aureus infections (24), this study has shown that Hcol strains produced more biofilm than the strains responsible for BJIs in our experimental conditions. Comparable results were obtained for GBS strains from vaginal colonization in pregnant women compared to strains isolated during infection of their newborn (22). Similar results were obtained for the pneumococci and S. aureus (25, 26). We may suggest that weak biofilm production seems to be correlated with increased pathogenicity.

The GBS BJI’s mode of contamination is unknown. The hypothesis could be that the contamination of joint is more likely haematogenic. Considering that the colonization of the host is the crucial first step during a haematogenic infection, patient could be colonized by strong biofilm capacity strains. Thus, having a continuous carriage could graft into bone joint. The search for rectal, vaginal or urinary colonization was not performed for these patients. A study to compare BJI GBS strains with colonization strains isolated from the same patient could bring elements to understand the mechanism.

Biofilm assay was carried out according to the existing protocols in the literature. The main difficulty of this work is to compare with other studies. Indeed, the methods of biofilm studies (conditions static or dynamic, flat or round plates, the starting inoculum, culture) and analysis vary in the literature (18, 21–23, 27, 28). It seems necessary to develop harmonization and standardization techniques of biofilm production in order to compare literature results. Dynamics studies seems also to be essential.

Environmental conditions may affect the ability of biofilm formation. Acid pH would increase the ability to form biofilm in strains belonging to CC17 (23, 27). Glucose seems globally to be more important than pH for the biofilm production in GBS strains (28). But glucose leads acidification of the environment during bacterial growth by release of lactic acid.

Furthermore, the biofilm was only studied after 24 h of incubation, but several studies show a greater ability to produce biofilm with a longer time (48h, 72h) (19). This could be explaining the weak biofilm production from BJI strains. Finally, as in other studies, we analyzed the main step of biofilm production: the adherence and immature biofilm formation (6).

Other virulence factors should exist but are not yet discovered to explain the difference in biofilm formation. Isogenic mutants, metabolic pathways studies, transcriptomic analyses, and bacteriophages detection are needed in order to advance in understanding the physiopathology of biofilm production. A study using the Whole Genome Sequencing on GBS could bring element for the identification of virulence factors.

In conclusion, our study revealed that GBS strains responsible for BJI appeared to produce less biofilm than Hcol strains, though they belonged to the same phylogenetic lineage and had the same adhesin and pili content.

The univariate analysis consisted in a Chi2 or Fisher test for the categorical data, depending on the case. A value of *P* < 0.05 was considered significant.

## MATERIALS AND METHODS

### Study design.

The study was designed as a multicenter, retrospective, observational study of adult patients suspected to have GBS BJI.

### Collection of clinical data and strains.

The CRIOGO (Centre de référence des infections ostéo-articulaires du Grand Ouest) study group has collected 77 GBS strains isolated from BJI, in patients with (*n* = 51) or without (*n* = 16) orthopedic device, among six French University Hospitals between January 2010 and December 2016. The Hospital center of Tours has supplied GBS human colonizing 57 strains (Hcol) isolated from the vaginas of asymptomatic women at prenatal screening (week 35–37).

Clinical characteristics were obtained from questionnaires. Collected data included sex, birth date, date and origin of the sample, clinical data, antibiotic therapy and relapse.

### Definition of BJI.

Infection was defined by the presence of one intraoperative (from joint, bone, or synovial fluid) sample positive in culture with a GBS. This definition takes into account the major infection criteria of the MusculoSkeletal Infection Society (MSIS) guidelines (29). Early infection occurs within 1 month after an invasive procedure, delayed infection between 1 and 12 months, and late infection after 12 months.

### Inclusion criteria.

Adult, GBS monobacterial BJI.

### Molecular characterization of GBS strains.

**(i) DNA extraction**. Bacterial genomic DNA was extracted and purified by conventional methods (30) and used as the template for PCR assays.

**(ii) MLST**. The strains were analyzed using MLST according to the procedure described by Jones et al. (31). The sequences of each new allele and the composition of new STs identified are available at http://pubmlst.org/sagalactiae/. Major lineages of GBS strains were determined by MLST.

### Detection of adhesin and pili genes in the genomes of GBS strains.

The strains were screened to determine the presence of adhesin and pili genes: *bsaB*, *bibA*, *lmb*, *scpB*, *fbsA* and *fbsB*, *hvgA*, *srr-1* and *srr-2*, *bca*, and pili PI-1, PI-2a, and PI-2b genes by using PCR. Primers are listed in Table S1 (12–17, 19). PCRs were performed with an Applied Biosystems 2720 apparatus, using 1 U Ampli *Taq* DNA polymerase from Applied Biosystems in a 25-μL 1× Gene Amp buffer containing 1.5 mM MgCl_2_, 200 μM each deoxynucleoside triphosphate, 500 μM each primer, and 20 ng of genomic DNA. Cycling conditions were as follows: 1 cycle of 10 min at 94°C; 35 cycles of 1 min at 94°C, 1 min at primers temperature melting (Table S1), and 1 min per kb at 72°C; and a final extension of 10 min at 72°C. PCR products were separated in 1% agarose gels for 1 h at 10 V per cm of gel.

### Biofilm assay.

GBS strains were grown overnight and diluted 20-fold in Todd Hewitt (TH) broth (Sigma-Aldrich, St-Louis, Mo; Cat. n° T1438) supplemented with 1% glucose and adjusted to pH 6.5 at 35°C in 5% CO_2_ atmosphere. Then, cells were prepared for biofilm assay as described previously (18, 22). They were diluted to optic density (OD)_595nm_ = 0.5 from the overnight culture. Then, 200 μL culture aliquots were dropped into 96-well plates. Afterwards, plates were incubated under static conditions at 35°C in 5% CO_2_ atmosphere for 24 h, and bacterial sessile development was evaluated with 0.2% crystal violet coloration and dissolved by ethanol/acetone (80:20). After, biofilm formation was quantified by measuring absorbance of the solution at OD_595nm_. An untreated plate (TH without bacterial inoculation) was used as negative control. Each assay was performed in triplicate.

Specific Biofilm formation (SBF) was defined by OD_595nm_ treated plate/OD_595nm_ control plate. SBF < 1.5 represented a weak, SBF between 1.5 and 3 a moderate, and SBF> 3 a strong biofilm producer.

### Study endpoints.

Primary outcome was to define GBS BJI’s genotype favoring biofilm production. Secondary outcome was to study the difference biofilm production between GBS BJI or Hcol strains.

### Statistical analysis.

To find out the primary outcome, GBS strains were analyzed by their content in adhesin, pili and CC and compared according to their biofilm capacity. For the secondary outcome, GBS BJI and Hcol strains were compared on biofilm capacity production.

### Ethics statement.

This study was approved by the ethics review boards of each institution. Patient consent was waived because the study was a retrospective analysis of routinely collected data, and there was no study-related intervention.
