# Supplementary material for: Genotypic Characterization and Biofilm Production of Group B Streptococcus Strains Isolated from Bone and Joint Infections
Source: Microbiol Spectr. 2022 Mar 31;10(2):e02329-21. doi: 10.1128/spectrum.02329-21 (PMC9045227; doi:10.1128/spectrum.02329-21)
Supplement: SUPPLEMENTAL FILE 1 — Supplemental material. Download SPECTRUM02329-21_Supp_1_seq4.pdf, PDF file, 0.04 MB [file spectrum02329-21_supp_1_seq4.pdf]

Table S1. PCR primers used to detect adhesin and virulence genes in the genomes of the 134 GBS isolates.

**Gene**	**5'-3' Left primer sequence**	**5'-3' Right primer sequence**	**amplicon size (bp)**	**Temperature melting (°C)**	**References**
PI-1	CTACCAACGGCCAAGCTATTTACC	TAGCCGCTTTTTCATTCTTTCTCC	394	53	Springman et *al.*, 2014
PI-2a	AACTCCCTATATTTGCAGGTTCAA	CGGGTGTAACGACTTTTATCTGAT	243	53	Springman et *al.*, 2014
PI-2b	GGGGGTAGGCTTAATGGCTTAT	TCCGGTTTAACTGTTCTGATTTGAT	519	53	Springman et *al.*, 2014
bsaB	ACCTGTGAACGCTAAAGCTG	GCTGACCACTTGTCACCTCT	120	55	Jiang et *al*., 2014
bibA	AATCGAAAACAACGTTGGAAAG	AAACCAGGCTTCATCAGTCATT	630	53	Santi et *al*., 2007
srr1	CACTCTTAGGCCTGGAACAACA	TAGATTCCCAAGTCCTGATGC	1500	48	Wang et *al.*, 2014
srr2	TCACGCAAAGTTCGAGTTAAAA	AGATTTAGTAGCTCCTAA	1500	53	Wang et *al.*, 2014
lmb	CCCAAACAGCCTACGCAAG	TGCCTGCACCTGATTGGATC	118	53	Al Safadi et *al.*, 2010
scpB	TGTGACAGAAGACACTCCTG	CGTCATCTGCTACTGTTTCTCC	140	55	Al Safadi et *al.*, 2010
fbsA	CAACTTATAGGGAAAAATCCAC	AGTTAACATCGGTCTATTAGC	144	55	Al Safadi et *al.*, 2010
fbsB	GCGATTGTGAATAGAATGAGTG	ACAGAAGCGGCGATTTCATT	149	55	Al Safadi et *al.*, 2010
hvgA	ATACAAATTCTGCTGACTACCG	GTAAAATCCTTCCTGACCATTCC	201	55	Tazi et *al.*, 2010
bca	ACGACTTTCTTCCGTCCACTTAGG	TAACAGTTATGATACTTCACAGAC	535	53	Li *et al.*, 1999
